# Wild fish consumption and latitude as drivers of vitamin D status among Inuit living in Nunavik, northern Québec

**DOI:** 10.1017/S1368980024000491

**Published:** 2024-02-22

**Authors:** Matthew Little, Meghan Brockington, Amira Aker, Tiff-Annie Kenny, Federico Andrade-Rivas, Pierre Ayotte, Mélanie Lemire

**Affiliations:** 1 School of Public Health and Social Policy, University of Victoria, 3800 Finnerty Rd, Victoria, BC, Canada; 2 Department of Population Medicine, University of Guelph, Guelph, ON, Canada; 3 Axe santé des populations et pratiques optimales en santé, Centre de recherche du CHU de Québec-Université Laval, Québec, QC, Canada; 4 Département de médecine sociale et préventive, Université Laval, Québec, QC, Canada; 5 Institut de biologie intégrative et des systèmes, Université Laval, Québec, QC, Canada; 6 Centre de toxicologie du Québec, Institut national de santé publique du Québec, Québec, QC, Canada

**Keywords:** Vitamin D, Nutrition, Epidemiology, Indigenous health, Inuit health, Wild fish

## Abstract

**Objective:**

To measure vitamin D status and estimate factors associated with serum 25-hydroxyvitamin D (25(OH)D) in Nunavimmiut (Inuit living in Nunavik) adults in 2017.

**Design:**

Data were from *Qanuilirpitaa*? 2017 Nunavik Inuit Health Survey, a cross-sectional study conducted in August–October 2017. Participants underwent a questionnaire, including an FFQ, and blood samples were analysed for total serum 25(OH)D.

**Setting:**

Nunavik, northern Québec, Canada.

**Participants:**

A stratified proportional model was used to select respondents, including 1,155 who identified as Inuit and had complete data.

**Results:**

Geometric mean serum vitamin D levels were 65·2 nmol/l (95 % CI 62·9–67·6 nmol/l) among women and 65·4 nmol/l (95 % CI 62·3–68·7 nmol/l) among men. The weighted prevalence of serum 25(OH)D < 75 nmol/l, <50 nmol/l <30 nmol/l was 61·2 %, 30·3 % and 7·0 %, respectively. Individuals who were older, female, lived in smaller and/or more southerly communities and/or consumed more country (traditional) foods were at a reduced risk of low vitamin D status. Higher consumption of wild fish was specifically associated with increased serum 25(OH)D concentration.

**Conclusion:**

It is important that national, regional and local policies and programs are in place to secure harvest, sharing and consumption of nutritious and culturally important country foods like Arctic char and other wild fish species, particularly considering ongoing climate change in the Arctic which impacts the availability, access and quality of fish as food.

Vitamin D, which comprises a group of fat-soluble seco-sterols, including ergocalciferol-D2, cholecalciferol-D3 and alfacalcidol, has received increasing interest in recent decades for its physiological functions and global epidemiology. The biological actions of vitamin D (which is inactive by itself) are due to the functions of the metabolite calcitriol and include regulation of serum calcium and phosphate homoeostasis, and consequently bone development and the maintenance of bone health^(1,2)^. Deficiency of vitamin D can cause bone disorders, including rickets and osteomalacia^(3)^. Other identified vitamin D functions include the regulation of cell proliferation and anti-proliferation, cell differentiation and apoptosis, as well as immunomodulatory effects^(4)^. Epidemiological evidence has further linked poor vitamin D status to muscle weakness and propensity to falls^(1)^, as well as increased risk of certain cancers, autoimmune diseases (e.g. type 1 diabetes and multiple sclerosis), cardiometabolic disease, infections, asthma, active tuberculosis and all-cause mortality^(5)^. A unique characteristic of vitamin D as a nutrient is that it can be ingested from dietary sources and also synthesised by the human body through skin exposure to ultraviolet B (UVB) radiation^(2)^.

Previous evidence has indicated that Indigenous populations living in northern Canada, including Inuit, experience higher prevalence of vitamin D deficiency and associated health concerns (e.g. rickets) than reference populations living in southern regions^(6–8)^. Populations living at northern latitudes experience reduced solar UVB radiation exposure, reducing opportunities for vitamin D synthesis (see online supplementary material, Supplementary Figure 1)^(9)^. At present, however, there is limited evidence on how dietary intake and vitamin D ingestion may compensate for limited UVB radiation exposure and affect individual-level vitamin D status. This is particularly important in the context of Inuit food systems, in which country foods – wild foods harvested from the terrestrial and marine environments that are often rich in vitamin D – play an important role^(10)^. Indeed, it is well-documented that Inuit communities rely on country foods to sustain food security, cultural continuity, livelihoods, intergenerational knowledge transfer, nutrition and health^(11,12)^. However, while country foods are often strongly culturally preferred, Inuit communities have undergone a dietary transition due to colonisation, globalisation and economic and social development^(12)^. Market foods (especially those of low nutritional quality) represent a large fraction of contemporary diets on the basis of dietary energy^(13)^, but contribute only modestly to intake of several key nutrients, including vitamin D^(14)^. As such, vitamin D intake, insufficiency and deficiency are likely driven by a combination of socio-cultural, geographical and dietary factors and require complex multivariable methods to identify opportunities for public health communication and intervention to improve nutritional health across Inuit Nunangat (the Inuit homelands comprising the Inuvialuit Settlement Region, Nunavut, Nunavik and Nunatsiavut).

Very few previous studies have evaluated vitamin D status among Nunavimmiut (Inuit living in Nunavik, northern Québec). In 2004, the *Qanuippitaa*? (How are we?) Nunavik Inuit Health Survey (Q2004) highlighted that a large proportion of Inuit adults (83 %) reported inadequate vitamin D intake using a 24-h dietary recall but did not directly measure vitamin D status among participants^(15)^. Given this context, the primary objective of this study is to measure vitamin D status and estimate the current prevalence and risk factors associated with vitamin D status in Nunavimmiut adults. In particular, we explore associations between vitamin D status and geographical factors (e.g. latitude), diet and socio-demographic factors, in an attempt to ascertain the primary drivers of serum vitamin D. The findings of our research may inform public health efforts to achieve improvements in vitamin D status and health among Nunavimmiut.

## Methods

### Study population

This study used data from the *Qanuilirpitaa*? (How are we now?) 2017 Nunavik Inuit Health Survey (Q2017), a cross-sectional study conducted in fourteen communities across Nunavik. For a complete description of the survey methods, see the methodological report^(16)^. Briefly, the health survey relied on a participatory approach grounded in partnerships between major Nunavik organisations (e.g. the Nunavik Regional Board of Health and Social Services, Makivik Corporation), the two hospitals in the region, the Institut national de santé publique du Québec and academic researchers. This approach was guided by OCAP® principles (Ownership, Control, Access and Possession), which are important terms of reference for participatory research by and for Indigenous People^(17)^. Ethical approval was received from the Comité d’éthique de la recherche du Centre Hospitalier Universitaire de Québec–Université Laval (no. 2016-2499). The objective of Q2017 was to provide information on various aspects of the physical and psychosocial health of Nunavimmuit. The target population was all permanent residents of Nunavik aged 16 years and older who were registered beneficiaries of the James Bay and Northern Québec Agreement, an Indigenous land claims agreement that affirms Indigenous rights and dictates governance structures in the region. A stratified proportional model was used to select respondents, with stratification based on sex, age group (16–19, 20–30 and 31+ years old) and community. Informed written consent was provided by all respondents. All surveyed individuals identifying as non-Inuit were excluded from this study.

### Data collection

**Fig. 1:**
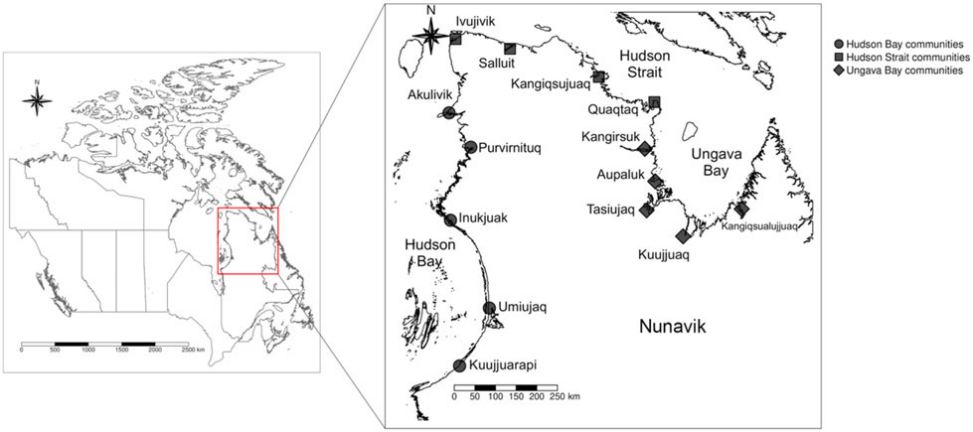
Map of Canada (left) and Nunavik (right), including the fourteen communities where participants were recruited for the *Qanuilirpitaa*? (How are we now?) 2017 Inuit Health Survey and their corresponding ecological regions (Ungava Bay, Hudson Strait, Hudson Bay). Map developed using information from the Database of Global Administrative Areas (GADM) and the Partenariat Données Québec under the Creative Commons Licence 4·0. Coordinate reference system: WGS84/EPSG: 6326. Produced in R 4·1·1 using the *tmap*, *terra* and *sf* packages

Data collection occurred from 19 August 2017 to 5 October 2017. The *Amundsen*, an icebreaker converted to an Arctic research vessel and operated by the Canadian Coast Guard, was used as a floating clinic for the purposes of the study. The *Amundsen* travelled to fourteen communities in Nunavik (Fig.1), and a team of researchers recruited participants, who were invited on board the ship for data collection. Communities in Nunavik range from approximately 200 residents to over 3,000 residents and were categorised as small (population <1,500) and large (population ≥1,500) communities for the purposes of data collection and analysis.

Each participant underwent a questionnaire that collected information on psychosocial health, physical health, socio-demographic characteristics and traditional harvesting practices. An FFQ collected information on the frequency of consumption of sixty-five food items over the three preceding months, including both country foods and market foods. Food insecurity was assessed using an adapted version of the United States Department of Agriculture Household Food Security Survey Module^(18)^, and respondents were categorised into one of four categories: food secure; marginal food insecurity; moderate food insecurity or severe food insecurity^(19)^. Questionnaires were administered by a team of twenty-four interviewers, twelve of whom were Inuit. Clinical tests included anthropometric measurements, including height and weight. Research nurses collected blood samples, which were obtained through venipuncture and placed in clot activator vacutainers. Samples were processed within 90 min on board the *Amundsen*. Vacutainers were left standing at room temperature for 30 min to allow complete coagulation, then were centrifuged at 2000× g for 10 min. The serum was then transferred into a 4·5 ml polypropylene tube for storage at –20°C until time of analysis.

### Vitamin D status

Serum 25-hydroxyvitamin D (25(OH)D) levels were measured using an electrochemiluminescence binding assay on a MODULAR ANALYTICS e170 analyser from Roche Diagnostics GmbH (Mannheim, Germany) at the Institut universitaire de cardiologie et de pneumologie de Québec (Quebec, QC, Canada). This assay received certification through the Centers for Disease Control and Prevention Vitamin D Standardization-Certification Program (CDC VDSCP) for successfully meeting the performance criterion of ±5·0 % mean bias when compared with CDC reference measurement procedure and an overall imprecision of <10 %^(20)^. The limit of detection of the method is 7·5 nmol/l. Data from the manufacturer indicate repeatability CV (%) values of 6·8 %, 5·2 %, 3·9 % and 2·2 % for pooled plasma sera controls containing 20·9, 39·5, 70·8 and 174 nmol/l, respectively. This method has been standardised against a liquid chromatography tandem MS reference method^(21)^. Participants were categorised based on serum 25(OH)D concentration into one of four categories: <30 nmol/l (severe vitamin D deficiency), ≥30 nmol/l and <50 nmol/l (vitamin D deficiency), ≥50 nmol/l and <75 nmol/l (low vitamin D status) or ≥75 nmol/l (vitamin D sufficiency)^(22,23)^.

### Developing the dietary profiles and frequency of food group consumption

The FFQ measured consumption frequency of each item over the previous 3 months but did not collect information on serving size. Due to the high collinearity between food intake variables, we used dietary profiles (based on market and country foods) and country food consumption profiles (using only country food variables) derived using latent profile analysis by Aker and colleagues^(24)^. Briefly, the intention of this process was to identify the types of food consumption profiles in the population. Outliers were removed by including only those foods consumed by at least 75 % of the study population. The best model was selected based on the optimal number of *k* unobserved profiles that described the relationship between variables based on Akaike’s information criterion, Bayesian information criterion and entropy values. This process established four profile groups that captured overall dietary behaviours (‘low consumption’, ‘country food dominant’, ‘market food dominant’ and ‘diverse consumption’) and four groups that captured the frequency of country food consumption (‘non-consumers’, ‘low consumers’, ‘medium consumers’ and ‘high consumers’)^(24)^.

### Statistical analysis

All statistical analyses were performed in Stata® Version 17.0 (Statacorp, College Station). The distribution of serum 25(OH)D levels was assessed using a histogram and a Shapiro–Wilk test for normality. Descriptive statistics were calculated, including geometric mean serum 25(OH)D levels and prevalence of serum 25(OH) D concentration range by category of age, sex, education, ecological region, community size and latitude, food security status, BMI, hunting and traditional harvesting activities and food profile groups. T-tests and Kruskal–Wallis ANOVA tests with Bonferroni pairwise comparisons were used to assess differences serum 25(OH)D levels between groups at a significance of *P* < 0·05. Multicollinearity was assessed using the variation inflation factor; variables were considered collinear if they had a variation inflation factor of >5.

Serum 25(OH)D concentration was not normally distributed after assessment using a histogram and a Shapiro–Wilk test. We therefore log-transformed this variable prior to building regression models following a Box Cox analysis to determine the best transformation. We performed multiple linear regression analyses to derive regression coefficients with log-transformed serum 25(OH)D concentrations as the dependent variable and various socio-demographic, lifestyle and dietary factors as independent variables. First, we assessed associations between potential predictors (based on existing literature) and log-transformed serum 25(OH)D concentrations while controlling only for age, sex, month of blood draw (August, September or November) and latitude of home community (degrees North). Next, we developed a fully adjusted linear regression model, which included age, sex, education, income, community size (large or small), latitude of residence (degrees N), smoking (none, occasionally and frequently), month of blood draw (August, September and October), traditional harvesting activities (yes/no) and food profile groups. Age and latitude of home community were continuous variables, while all other variables were categorical. Since the ‘high’ country food consumers were a relatively small (*n* 101) group, the ‘medium’ and ‘high’ country food consumers were aggregated into one group. Model assumptions were tested by assessing residuals for normal distribution and heteroscedasticity using a scatterplot of standardised residuals *v*. fitted values.

Next, we performed a multinomial logistic regression to investigate the association between potential predictors and serum 25(OH)D categories <30 nmol/l and ≥30 nmol/l but <50 nmol/l, using serum 25(OH)D ≥ 50 nmol/l as the referent category. These categories were selected based on the cut-offs for vitamin D sufficiency promoted by the Institute of Medicine^(2)^. Crude and adjusted relative risk ratios and 95 % CI were calculated for each variable of interest. As above, variables that were hypothesised *a priori* as potential predictors or confounders based on existing literature were retained in the multinomial model regardless of their association with the outcome^(25)^. We tested interactions between multiple variables, including age, sex, community size, traditional harvesting activities and food profile groups. Interaction variables were excluded from the model if they were not associated with either outcome at *P* < 0·05. A generalised Hosmer–Lemeshow goodness-of-fit-test was used to evaluate the fit of the final model.

Finally, we used linear regression analyses to assess associations between frequency of consumption of different categories of country foods and market foods (over the 3 months prior to the survey administration) and serum 25(OH)D concentration. To aggregate items into food groups, we standardised the consumption of each item to the frequency per month taking the average within each response option. For example, a response of 2 (item consumed 1–3 times per month) was categorised as two times per month. The two exceptions were a response of 1 (item consumed never or less than once a month), which was categorised as 0·5 times per month, and 7 (item consumed four times or more a day), which was categorised as 120 times per month (i.e. four times per day for thirty days per month on average). In calculations, it was assumed that there were 4 weeks and 30 d per month. Once items were standardised as frequency per month, we summed them into the following food categories: wild fish, marine mammals, shellfish, land mammals, wild birds, wild berries, store-bought meats, canned fish, milk products, fruits and fruit juices and vegetables. We assessed associations between each food category/item and serum 25(OH)D concentration while adjusting for age, sex, community latitude and month of data collection (August/September/October). Next, we assessed associations between each food category and serum 25(OH)D concentration while adjusting for the same list of variables in addition to the frequency of consumption of all other food categories.

Due to the complex study design, all distributions and regression analyses were weighted using survey weights with the svyset function in Stata 17 to account for probability of being selected into each stratum and the non-response rate. Survey weights were calculated using age, sex and ecological region distribution to produce a representative population-level estimate. Variance of estimates (95 % CI) were calculated using the bootstrap (re-sampling with replacement) balanced repeated replication (brr) method using 500 sets of bootstrap weights^(16,26)^.

## Results

**Fig. 2:**
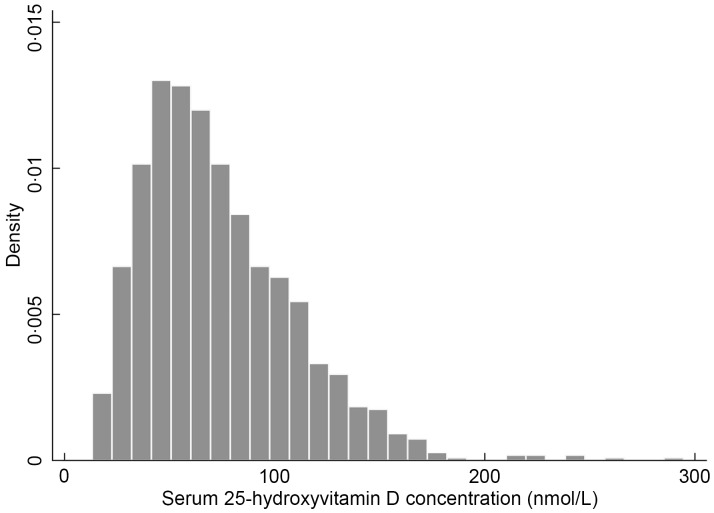
Unweighted frequency distribution of serum 25-hydroxyvitamin D levels in a sample (*n* 1,155) of Nunavimmiut recruited for the *Qanuilirpitaa*? (How are we now?) 2017 Inuit Health Survey

In total, 1,326 participants were involved in Q2017. Almost all (1,325) submitted a blood sample that was assessed for serum 25(OH)D levels. Of these, 1,155 identified as Inuit and completed an FFQ and were included in descriptive and multivariable analyses. The total response rate for Q2017 was 31 % for people aged 16–30 years and 42 % for people aged 31 years and over after accounting for non-contacts, refusal at time of recruitment and ‘no-shows’ on the day of data collection^(16)^. Serum 25(OH)D levels showed non-normal distribution (Shapiro–Wilk test score *P* < 0·001) (Fig.2). Geometric mean serum 25(OH) levels were 65·2 nmol/l (95 % CI 63·0–67·6 nmol/l) among women and 65·4 nmol/l (95 % CI 62·3–68·7 nmol/l) among men. Median and arithmetic mean serum 25(OH)D levels were 66·2 and 73·5 nmol/l, respectively. The weighted prevalence of serum 25(OH)D < 75 nmol/l, <50 nmol/l, <30 nmol/l was 61·2 %, 30·3 % and 7·0 %, respectively.

Serum 25(OH)D concentrations and prevalence of serum 25(OH)D categories varied by several socio-demographic and geographic characteristics (Table 1). Specifically, serum 25(OH)D concentrations increased, and the prevalence of serum 25(OH)D < 50 nmol/l and ≥30 nmol/l or <30 nmol/l decreased, among individuals who were older, had less education, earned $15 000 per year or more, lived in smaller communities, at lower latitudes and/or in Hudson Bay or Ungava Bay (compared with Hudson Strait), did not smoke, participated in hunting, fishing and other traditional activities and consumed more country foods. There was no significant difference in serum 25(OH)D concentration or prevalence of vitamin D categories by sex, BMI and food security status.

**Table 1:** Geometric mean serum 25(OH)D level and weighted prevalence of vitamin D categories by socio-demographic, geographic and lifestyle characteristics in Nunavimmiut (Inuit living in Nunavik, Québec, Canada; *n* 1,155) participants in the Q2017 Health Survey

	*n*	Serum 25(OH) D levels geometric mean in nmol/l	95 % CI	*T*-test/Kruskal-Wallis *P*-value	% serum 25(OH)D < 75 and *≥50 nmol/l* (*n* 344)	% serum 25(OH)D < 50 and *≥30 nmol/l* (*n* 256)	% serum 25(OH)D < 30 nmol/l (*n* 79)
**Age**							
16–19	203	52·5	49·7, 55·6	<0·001	36·6	38·0	6·8
20–39	466	56·3	54·1, 58·7		36·9	26·4	9·3
40–59	369	75·0	71·6, 78·6		25·4	17·3	5·7
60+	117	110·9	103·0, 119·3		10·0	3·3	0·9
All ages	1,155	65·3	63·5, 67·2		30·9	22·2	6·8
**Sex**							
M	389	65·4	62·3, 68·7	0·90	30·6	25·3	6·9
F	766	65·2	63·0, 67·6		31·1	21·2	7·1
**Education**							
Grade 6 or less	107	86·4	77·9, 95·8	<0·001	17·3	12·3	6·8
Some secondary school (Grade 7–10)	693	62·4	60·2, 64·8		30·8	23·9	8·4
Secondary school (Grade 11) or higher	330	63·5	60·4, 66·7		36·9	26·9	4·6
**Income (per year)**							
<$15 000	402	60·1	57·3, 63·0	<0·001	30·9	27·0	8·5
$15 000–$25 000	198	70·2	65·4, 75·5		24·3	21·3	5·1
$25 000–$60 000	242	67·6	63·6, 71·8		34·7	20·8	5·8
>$60 000	145	70·9	65·5, 76·6		27·1	24·6	6·8
**Community size**							
Large (pop. ≥1500)	583	58·7	56·3, 61·1	<0·001	32·7	25·7	9·4
Small (pop. <1500)	572	72·8	70·1, 75·6		25·8	20·0	3·7
**Region**							
Hudson Bay	376	68·0	64·8, 71·4	<0·001	28·1	20·8	5·1
Hudson Strait	277	52·4	49·3, 55·6		32·6	32·9	15·7
Ungava Bay	502	71·5	61·7, 74·5		33·0	19·6	3·1
**Community latitude by tertile**							
Tertile 1 (55·3–58·5°N)	409	63·7	60·8, 66·8	<0·001	35·1	28·1	5·3
Tertile 2 (58·6–60·1°N)	407	74·8	71·6, 78·2		30·9	14·0	3·5
Tertile 3 (60·2–62·4°N)	363	57·1	54·0, 60·3		25·6	27·8	13·0
**Interview/blood draw month**							
August	264	64·9	61·2, 68·8	0·11	33·5	24·7	6·0
September	785	66·0	63·7, 68·4		28·6	21·9	8·1
October	106	61·0	56·1, 66·3		39·1	29·0	2·1
**Food security status** *							
Food secure	249	66·4	62·4, 70·7	0·48	33·6	18·6	8·9
Marginal food insecurity	125	66·2	60·9, 72·0		24·1	23·0	4·0
Moderate food insecurity	523	65·1	62·4, 67·8		31·1	24·8	6·0
Severe food insecurity	204	62·5	58·4, 66·8		32·8	25·5	7·8
**BMI**							
<18·5 kg/m	26	60·4	46·6, 78·3	0·35	28·3	10·9	18·7
≥18·5–<25 kg/m^2^	463	64·0	61·1, 67·0		27·0	27·6	7·3
≥25–<30 kg/m^2^	303	68·4	64·9, 72·0		34·5	17·9	4·7
≥30 kg/m^2^	363	64·8	61·6, 68·3		33·6	22·4	7·7
**Smoking**							
Not at all	252	71·1	66·8, 75·6	0·003	29·4	18·3	7·4
Occasionally	96	68·5	63·0, 74·4		29·7	26·5	4·7
Daily	807	63·3	61·1, 65·5		31·4	24·3	7·1
**Harvesting or traditional activities**							
Did not participate in harvesting or traditional activities	119	58·1	53·1, 63·7	0·003	31·9	25·8	11·8
Participated in harvesting or traditional activities	1,033	66·3	64·3, 68·3		30·6	23·1	6·4
**Fishing**							
Did not fish at least once per month every season for past year	918	64·0	62·0, 66·2	0·005	31·8	23·2	8·1
Fished at least once per month every season for the past year	277	70·7	66·6, 75·1		29·5	23·4	3·2
**Hunting**							
Did not hunt at least once per month every season for past year	605	62·9	60·4, 65·4	0·011	31·2	24·4	8·4
Hunted at least once per month every season for the past year	542	68·0	65·2, 70·8		28·4	22·6	5·7
**Overall dietary profile**							
Market food dominant	489	62·6	59·9, 65·4	0·014	31·8	24·3	8·4
Country food dominant	116	72·9	66·7, 79·6		26·1	22·9	3·5
Diverse consumption	278	66·1	62·6, 69·9		31·6	21·2	7·0
Low consumption	272	66·5	62·6, 70·6		31·2	24·0	6·4
**Country food dietary profile**							
None	368	58·8	55·9, 62·0	<0·001	30·7	28·0	10·3
Low	520	67·6	64·8, 70·6		31·4	20·2	6·8
Moderate	171	68·2	63·7, 73·1		34·5	20·8	3·8
High	101	74·2	60·6, 72·8		24·5	25·0	3·1

25(OH)D, 25 hydroxyvitamin D.

* Food security status based on an adapted version of the USDA Household Food Security Survey Module and determined using the PROOF food security categories (Tarasuk et al., 2013).

No variables were multicollinear according to our analysis of variation inflation factor (results not shown). After multivariable adjustment, individual income of $15 000–$25 000 per year (compared with <$15 000 per year), living in a small community, living in a more southerly community, participating in traditional and harvesting activities and low or moderate/high country food consumption (compared with no country food consumption) were associated with higher vitamin D status (Table 2). Blood drawn in September (in comparison with August) was associated with higher vitamin D status when controlling for age, sex and community latitude, but this association did not reach significance after adjusting for all predictors. Smoking was not associated with serum 25(OH)D concentrations in either linear regression model. No concerns were identified after assessing normality of residuals and homoscedasticity of the final fully adjusted model (results not shown). No interaction variables were associated with the outcome at *P* < 0·05 and were thus not included in the final model. The adjusted *R*-squared value was 0·32.

**Table 2:** Associations between potential predictors and log-transformed serum 25(OH)D concentrations in Nunavimmiut (Inuit living in Nunavik, Québec, Canada; *n* 1,155) participants in the Q2017 Health Survey

Predictor (unit) (referent)	Difference in serum 25(OH)D (nmol/l)					
	Adjusted for age, sex, community latitude and month of blood draw*			Adjusted for all predictors†		
	Coefficient	BRR s e	*P*-value	Coefficient	BRR s e	*P*-value
**Age (years)**	0·097	0·09	<0·001	0·097	0·007	<0·001
**Sex**						
(F)						
M	0·025	0·02	0·31	−0·042	0·03	0·12
**Education**						
(Grade 6 or less)						
Some secondary school (Grade 7–10)	−0·067	0·06	0·23	−0·048	0·06	0·41
Secondary school (Grade 11) or higher	−0·091	0·06	0·11	−0·061	0·07	0·34
**Income (per year)**						
(<$15 000)						
$15 000–$25 000	0·079	0·04	0·05	0·10	0·04	0·018
$25 000–$60 000	−0·025	0·04	0·49	−0·010	0·04	0·80
>$60 000	−0·07	0·05	0·15	−0·029	0·05	0·59
**Community size**						
(Large community)						
Small community	0·16	0·03	<0·001	0·14	0·04	<0·001
**Community latitude (degrees** ***n*** **)**	−0·054	0·009	<0·001	−0·044	0·01	<0·001
**Interview/blood draw month**						
(August)						
September	0·14	0·04	<0·001	0·019	0·05	0·67
October	−0·019		0·70	0·036	0·05	0·48
**Smoking**						
(Not at all)						
Occasionally	0·081	0·06	0·16	0·084	0·06	0·19
Daily	0·035	0·04	0·34	0·011	0·04	0·79
**Harvesting or traditional activities**						
(No)						
Yes	0·13	0·05	0·008	0·12	0·05	0·017
**Country food dietary profile**						
(None)						
Low	0·14	0·03	<0·001	0·12	0·03	0·001
Moderate and high	0·23	0·04	<0·001	0·19	0·04	<0·001

25(OH)D, 25 hydroxyvitamin D; BRR, balanced repeated replication; Q2017 Health Survey, Qanuilirpitaa? 2017 Inuit Health Survey; se, standard error

* Adjusted for age (years), sex, month of blood draw and community latitude (degrees North) using multivariable linear regression analysis incorporating sampling weights and balanced repeated replication standard error estimates

† Adjusted for age (years), sex, month of blood draw and community latitude (degrees North), education, income, community size, interview/blood draw month (August, September or October), smoking, participation in harvesting or traditional activities and country food consumption profile using multivariable linear regression analysis incorporating sampling weights and balanced repeated replication se estimates

In the adjusted multinomial logistic regression model (Table 3), individuals who were older, female, lived in smaller and/or more southerly communities and/or consumed more country foods were at a lower risk of serum 25(OH)D < 50 nmol/l and ≥30 nmol/l (compared with serum 25(OH)D ≥ 50 nmol/l). Those living at more southerly latitudes and/or in smaller communities, as well as those who consumed more country foods, were also at lower risk of serum 25(OH)D < 30 nmol/l (compared with serum 25(OH)D ≥ 50 nmol/l). No interaction variables were associated with either outcome at *P* < 0·05 and were thus not included in the final model. The Hosmer–Lemeshow test showed the final adjusted model had a strong goodness of fit.

**Table 3:** Associations between serum 25(OH)D categories and socio-demographic, geographic and lifestyle characteristics among Nunavimmiut (Inuit living in Nunavik, Québec, Canada; *n* 1,155) participants in the Q2017 Health Survey

Variable	Relative risk ratio (fully adjusted)*	95 % CI	*P*-value
**Serum 25(OH)D > 50 nmol/l (base outcome)**			
**Serum 25(OH)D > 30 nmol/l ≤ 25(OH)D ≤ 50 nmol/l**			
**Age**			
(16–19)			
20–39	0·63	0·36–1·13	0·12
40–59	0·33	0·17–0·63	0·001
60+	0·06	0·02–15·4	0·48
**Gender**			
(F)			
M	1·711	1·20–2·44	0·003
**Education**			
(Grade 6 or less)			
Some secondary school (Grade 7–10)	1·36	0·47–3·94	0·57
Secondary school (grade 11) or higher	1·70	0·55–5·33	0·36
**Income (per year)**			
(<$15 000)			
$15 000–$25 000	0·76	0·44–1·34	0·35
$25 000–$60 000	0·82	0·46–1·49	0·52
>$60 000	1·28	0·63–2·59	0·49
**Community size**			
(Large community)			
Small community	0·60	0·37–0·99	0·047
**Community latitude (degrees N)**	1·14	0·97–1·32	0·11
**Interview/blood draw month**			
(August)			
September	1·00	0·56–1·79	0·99
October	1·13	0·55–2·33	0·73
**Smoking**			
(None)			
Occasionally	0·91	0·39–2·11	0·82
Daily	0·94	0·57–1·53	079
**Harvesting or traditional activities**			
(Yes)			
No	1·13	0·55–2·32	0·74
**Country food dietary profile**			
(Not at all)			
Low	0·62	0·40–0·94	0·024
Moderate or high	0·55	0·32–0·97	0·038
**Serum 25(OH)D < 30 nmol/l**			
**Age**			
(16–19)			
20–39	2·18	0·64–7·41	0·21
40–59	0·56	0·17–1·91	0·36
60+	0·07	0·002–3·71	0·78
**Gender**			
(F)			
M	1·07	0·52–2·18	0·57
**Education**			
(Grade 6 or less)			
Some secondary school (grade 7–10)	0·65	0·009–48·5	0·85
Secondary school (grade 11) or higher	032	0·004–25·0	0·60
**Income (per year)**			
(<$15 000)			
$15 000–$25 000	0·42	0·12–1·49	0·18
$25 000–$60 000	0·67	0·26–1·69	0·39
>$60 000	0·99	0·27–2·36	0·87
**Community size**			
(Large community)			
Small community	0·30	0·13–0·68	0·004
**Community latitude (degrees N)**	1·71	1·26–2·31	0·001
**Interview/blood draw month**			
(August)			
September	1·1	0·40–2·96	0·86
October	0·79	0·006–102·5	0·92
**Smoking**			
(None)			
Occasionally	0·58	0·02–12·42	0·89
Daily	0·92	0·36–2·37	0·87
**Harvesting or traditional activities**			
(Yes)			
No	2·19	0·80–6·04	0·13
**Country food dietary profile**			
(Not at all)			
Low	0·40	0·18–0·87	0·021
Moderate and high	0·21	0·07–0·66	0·008

25(OH)D, 25 hydroxyvitamin D; Q2017 Health Survey, Qanuilirpitaa? 2017 Inuit Health Survey; BRR, balanced repeated replication.

* Adjusted for age (years), sex and community latitude (degrees North), education, income, community size, interview/blood draw month (August, September or October), smoking, participation in harvesting or traditional activities and country food consumption profile using weighted multivariable multinomial logistic regression analysis with BRR se estimates.

We specifically investigated associations between frequency of consumption of country foods and market foods and serum 25(OH)D status. Higher consumption of wild fish (including the sum of average monthly consumption of air-dried fish, Arctic char, lake trout, brook trout, sea trout, salmon, pike or walleye and ‘other’ fish) was associated with serum 25(OH)D concentration in partially and fully adjusted models (Table 4). While fruits and fruit juice consumption were negatively associated with vitamin D status in the partially adjusted model, this association was NS at *P* < 0·05 after controlling for other food items.

**Table 4:** Associations between frequency of country food and market foods and serum 25-hydroxyvitamin D (25(OH)D) concentrations among Nunavimmiut (Inuit living in Nunavik, Québec, Canada; *n* 1,155) participants in the Q2017 Health Survey

Frequency of consumption of food item (approximate times per month, on average)*	Beta coefficient, partially adjusted**	95 % CI	*P* value	Beta coefficient, fully adjusted*†	95 % CI	*P* value
**Country foods**						
Wild fish†	0·15	0·04, 0·25	0·005	0·17	0·06, 0·28	0·003
Marine mammals‡	0·04	–0·007, 0·09	0·095	0·007	–0·06, 0·08	0·84
Shellfish§	0·25	–0·04, 0·54	0·092	0·19	–0·91, 0·47	0·19
Land mammals||	0·047	–0·001, 0·09	0·056	0·006	–0·06, 0·07	0·90
Wild birds¶	0·07	–0·04, 0·18	0·19	−0·004	–0·15, 0·14	0·95
Wild berries	0·07	–0·02, 0·15	0·13	0·016	–0·09, 0·12	0·77
**Market foods**						
Store-bought meats and eggs	−0·05	–0·10, 0·008	0·10	−0·05	–0·09, 0·12	0·18
Canned fish	−0·14	–0·50, 0·21	0·43	−0·16	–0·11, 0·02	0·40
Milk products	−0·009	–0·03, 0·03	0·96	0·013	–0·03, 0·06	0·57
Fruits and fruit juices	−0·05	–0·096, –0·012	0·012	−0·05	–0·11, 0·01	0·13
Vegetables	−0·03	–0·08, 0·008	0·12	−0·03	–0·09, 0·03	0·34

25(OH)D, 25 hydroxyvitamin D; Q2017 Health Survey, Qanuilirpitaa? 2017 Inuit Health Survey.

* In the 3 months prior to the survey.

† Includes dried fish, lake trout, brook or sea trout, salmon, Arctic char, pike or walleye and ‘other’ fish (e.g. Lake whitefish and sculpin).

‡ Includes beluga, seal and walrus products.

§ Includes mussels, scallops, clams and urchins.

|| Inclides caribou, polar bear and muskox.

¶ Includes ptarmigan, partridge and goose.

** Adjusted for age (years), sex, community latitude and month of data collection.

*† Adjusted for age (years), sex, community latitude, month of data collection and frequency of consumption of all food items.

## Discussion

Serum 25(OH)D is generally considered the best marker for determining vitamin D status. In this cross-sectional study of Nunavimmiut youth and adults (16 years of age and older), the weighted prevalence of serum 25(OH)D < 75 nmol/l was 61·2 %. This cut-off has recently been promoted as the threshold for low vitamin D status by several authors on the basis of emerging evidence on bone density and osteomalacia^(27)^. The weighted prevalence of serum 25(OH)D < 50 nmol/l was 30·3 %, which is considered vitamin D deficiency as defined by the Institute of Medicine and the cut-off commonly enforced in clinical practice^(2)^. Meanwhile, the weighted prevalence of serum 25(OH)D < 30 nmol/l was 7·0 %, which is considered severe vitamin D deficiency^(23,28)^. Blood samples were collected from mid-August to early October 2017 so levels represent those observed during the summer and early fall, when vitamin D concentrations are likely higher due to UVB exposure. Serum 25(OH)D levels among Nunavimmiut men (geometric mean: 65·4 nmol/l; arithmetic mean: 73·4 nmol/l) and women (geometric mean: 65·2 nmol/l; arithmetic mean: 73·5 nmol/l) were slightly higher than the Canadian general population (excluding Inuit Nunangat) in April–October of 2007–2009^(29)^ and substantially higher than those reported during the 2007–2008 International Polar Year (IPY) Survey, which was conducted between August and October in Nunavut, Inuvialuit Settlement Region and Nunatsiavut (see Table 5 for comparison with previous studies)^(30)^. To our knowledge, this is the highest mean serum 25(OH)D level recorded in any Inuit population, including Inuit living in Greenland^(31–33)^ and Alaska (Table 5)^(34,35)^. Prevalence of serum 25(OH)<50 nmol/l was slightly lower than the Canadian general population between 2009 and 2011 (32 %)^(36)^ and considerably lower than those reported in the 2007–2008 IPY Survey (42·2 %)^(30)^.

**Table 5:** Comparison of serum 25(OH)D concentrations among participants of Q2017 Health Survey with previous studies on the Canadian general population and Inuit from Canada, Greenland and Alaska

Study (year(s))	Study population	Season and year of data collection	Serum 25(OH)D arithmetic mean in nmol/l (variability)	Serum 25(OH)D geometric mean or median in nmol/l (variability)
**Qanuilirpitaa? Nunavik Inuit health survey (2017)**	Inuit (>15 years) (*n* 1,155)	August–October 2017	Men: 73·4 (69·8, 77·0); women: 73·5 (71·0, 76·1)	Men: 65·4 (62·3, 68·7)*; women: 65·2|| (63·0, 67·6)*
**Canadian health measures survey (2007–2009)**	Canadians (19–79 years) excluding Inuit Nunangat (*n* 3,562)	April–October 2007–2009	Men: 64·7 (61·0, 68·3) women: 72·0 (68·6, 75·4)	N/R
**International polar year survey Inuit health survey (2007–2008)**	Inuit (>18 years) in Nunavut, Inuvialuit Settlement Region and Nunatsiavut (*n* 2,207)	August–October 2007–2008	N/R	Men: 55·2¶ (34·7, 79·4)†; women: 51·7¶ (30·9, 78·6)†
**Inuit health in transition (2005–2010)**	Inuit (≥18 years) residing in nine towns and 13 villages (*n* 1,764)	Data collected during all months except July, November and December between 2005 and 2010	N/R	All participants: 43·4|| (41·8, 45·7)*
**Andersen et al., (2018)**	Inuit (50–59 years old) residing in Nuuk or Ammassalik District, Greenland (*n* 434)	N/R	N/R	All participants: 64¶ (25, 54) ‡
**Andersen et al., (2012)**	Inuit (30–49 years) in Saqqaq and Ilulissat, Greenland (*n* 64)	Summer 2002	N/R	All participants: 44·7¶ (36·4, 59·4)‡
**Frost and Hill (2008)**	Non-random sample of Alaska Natives (*n* 83)	2-y period between April 2005 and March 2007	All participants: 42·8 (±27·5)§	N/R

Q2017 Health Survey, Qanuilirpitaa? 2017 Inuit Health Survey; N/R, not reported.

* 95 % CI.

† Interquartile range.

‡ 25, 75 percentiles.

§ sd.

|| Geometric mean.

¶ Median.

Dermal production of vitamin D is determined by length of exposure to UVB light, latitude, season, skin pigmentation and use of protective clothing^(37,38)^. Higher solar zenith angles experienced at northern latitudes limit UVB light intensity during summer. However, spring and summer in Nunavik are often characterised by a relatively high index of sunlight, and exposure to UVB may be heightened due to reflection from snow and ice. In our study, latitude of residence was a strong predictor of vitamin D status, with those participants living in villages further north exhibiting lower serum 25(OH)D status and higher risk of serum 25(OH)D below 30 nmol/l. Further, participating in harvest and traditional (often land-based) activities was associated with higher vitamin D status, which may reflect exposure to UVB light during such activities. These findings suggest that sun exposure is an important predictor of vitamin D status in Nunavik, which is consistent with research conducted elsewhere in North America and Europe^(9)^. Unfortunately, no reliable observation data exist for UVB radiation in Nunavik. Such data would be useful to identify UVB exposure and potential for dermal vitamin D production in different regions of Nunavik.

Serum 25(OH)D levels were positively associated with age, and risk of vitamin D deficiency (serum 25(OH)D < 50 nmol/l and ≥30 nmol/l) was lower for individuals aged 40–59 years compared with those aged 16–19 years. Serum 25(OH)D levels were high, on average, for older age groups (60+), although risk of low vitamin D status was not significantly different for this group. Improved vitamin D status among older populations was not expected since older adults are often considered at risk for vitamin D deficiency due to decreased cutaneous synthesis^(39)^. Other studies among Inuit in the circumpolar north have shown no significant association between age and vitamin D status^(31)^. However, the positive association between age and vitamin D status is consistent with the most recent Canadian Health Measures Survey data, which showed that 41 % of those aged 20–39 years, 32 % of those aged 40–59 years and 25 % of Canadians aged 60–79 years exhibit vitamin D insufficiency or deficiency^(29)^. The higher prevalence of serum 25(OH)D < 50 nmol/l among young adults between the ages of 16 and 39 is concerning and indicates that screening and nutritional education and counselling should target this group. Mechanisms for associations between age and vitamin D status are uncertain but may include differences in diet quality, intake of supplements and fortified foods and differential exposure to UVB light due to participation in outdoor land-based activities.

Prior to colonial contact, Nunavimmiut diets consisted entirely of foods harvested from the land (*nunamiutait uumajuit*, ‘those that belong on the earth’), ocean (*tariurmiutait*, ‘those that belong in salt water’), intertidal zone (*tininnimiutait*, ‘those that belong on the shore’), ocean floor (*irqamiutait*, ‘those that belong on the bottom of the ocean’) and rivers and lakes (*imarmiutait uumajuit,* ‘those that belong to the water’)^(40)^. Research suggests Inuit across Inuit Nunangat are experiencing an ongoing a dietary transition, characterised by reductions in country foods and increased consumption in market foods. This transition is driven by social, economic, cultural and environmental changes driven by colonial processes and institutions^(12)^. Despite this, the harvest, preparation, sharing and consumption of country foods have important cultural and health benefits. Further, there is preliminary evidence that the dietary transition in Nunavik has slowed in recent years. Indeed, between the Q2004 and Q2017 surveys, country food consumption remained relatively constant in Nunavik, likely due to ongoing efforts to promote traditional land-based activities and the transmission of Inuit knowledge to younger generations^(41)^. Our research findings add to the robust body of literature indicating that country foods are an important source of essential nutrients, including vitamin D^(11,14,42)^. There is thus a need to continue supporting efforts that promote access to healthy country foods, including wild fatty fish such as Arctic char, through harvester support programs, community freezers and distribution infrastructure and intra- and inter-community food sharing^(43)^.

The RDA of vitamin D is 15 mcg/d for adults over 18 years old and 20 mcg/d for adults over 70 years old^(2)^. Fatty fish are known as some of the most concentrated dietary sources of vitamin D. All Arctic fish, but especially Arctic char, Arctic cod and lake trout, are excellent sources of vitamin D^(10,44)^. Indeed, estimated vitamin D concentrations range from 8·4–25·8 mcg/100 g in Arctic char and 19·70–22·3 mcg/100 g in lake trout, although such figures are based off very few samples and require validation^(10,45)^. Fish are an important country food across Nunavik; Arctic char are consumed by 83 % of Nunavimmiut and are the second most frequently consumed country food in Nunavik after caribou^(41)^. More frequent consumption of country foods was associated with higher serum 25(OH)D levels and reduced risk of low serum 25(OH)D status in our study. Findings correspond with previous research from Greenland showing that individuals consuming a diet comprising mainly of ‘traditional Inuit food items’ (i.e. country foods), including seal and whale, had considerably higher serum 25(OH)D concentrations in comparison with those who consumed mainly imported foods^(31)^. Our findings suggest that, among Nunavimmiut, this association is primarily driven by consumption frequency of wild fish, including traditionally air-dried fish (*pitsik*), as well as trout, salmon and Arctic char. Further research should clarify vitamin D concentrations in various Arctic fish organs (e.g. liver) and tissues and how dietary preferences among Inuit might affect vitamin D intake and sufficiency. Regardless, wild fish are culturally important, align with food preferences and should be promoted as healthy sources of vitamin D across Nunavik. Notably, older lake trout are known to accumulate elevated concentrations of mercury, so dietary recommendations should balance the benefits and risks of such foods^(42)^.

Marine mammal fat is also an excellent sources of vitamin D. For example, beluga fat contains approximately 9·3 mcg/100 g, and seal fat contains 75 mcg/100 g^(10,46)^. However, while marine mammal consumption frequency was correlated with vitamin D status, this association did not reach statistical significance. Vitamin D obtained through country foods may also explain the strong association between living in a small (*v*. large) community and improved vitamin D status, since smaller communities in Nunavik tend to harvest and consume more country foods^(41)^. Further, participating in harvest activities increases exposure to sunlight, further improving vitamin D status^(30)^. Market food sources of vitamin D include fortified foods such as milk, margarine and juice drinks. However, market foods were not associated with vitamin D status among Nunavimmiut in our study, indicating that store-bought products were less-important sources of vitamin D, aligning with previous assessments in Nunavik^(15)^ and elsewhere in Inuit Nunangat^(30)^. Despite this, geometric mean serum 25(OH)D among individuals who reported consuming no country foods was 58·8 nmol/l, which is above the 50 nmol/l cut-off for vitamin D clinical deficiency and underscores the importance of multiple sources of vitamin D, including UVB light exposure and supplements, which were not assessed in the present study.

The importance of country foods, and especially fish, to vitamin D intake and sufficiency among Inuit is well-documented; however, there are several research gaps that should be the focus of future investigation. The origins of vitamin D in Arctic fish remain unclear, although plankton have the capacity to make cholecalciferol by UVB irradiation of 7-dehydrocholesterol and are likely the primary source of vitamin D in fish food chains^(47)^. There is a need to explore the impacts of anthropogenic climate change on marine ecosystems, including the implications for fish species abundance and nutrient density of vitamin D^(48)^. Scenario-building research with First Nations communities along the British Columbia coastline suggests that climate-mediated declines in wild marine food availability may reduce dietary vitamin D by up to one-third^(49)^. While no such analyses are available for Nunavik, the impacts of climate change on food security and nutrition are potentially severe and may include increased risk of vitamin D deficiency^(50)^. Notably, farmed fish have 75 % lower concentrations of vitamin D compared with wild fish, underscoring the nutritional importance of subsistence fishing and the limitations of store-bought replacements if fish abundance or access to subsistence fishing declines due to climate change^(51)^. Finally, there is little knowledge of the effects of age, sex, season and latitude on vitamin D concentrations within country food species, including fish.

This study was strengthened by a large sample size, a complex survey design and robust statistical methods. However, there were several limitations to the research. Data were collected in 2017 so may not reflect current nutritional trends in the region. The cross-sectional survey design limits causal inference. The low response rate may be a substantial source of selection bias; however, survey weights were used to increase the representativeness of the sample in our analyses^(16)^. Blood samples were provided at only one point in time between mid-August and early October, limiting our ability to determine the impacts of seasonality (including seasonal dietary variations and temporal variations in UVB exposure) on vitamin D status and potentially confounding the association between location/latitude of residence and vitamin D status. While considered a stable metabolite, the half-life of serum 25(OH)D has been calculated as 10–199 d, with lower baseline serum 25(OH)D contributing to a longer observed half-life^(52)^. It is probable that serum 25(OH)D levels therefore differed depending on timing of blood sample collection due to the seasonal nature of UVB exposure and country food consumption, although we controlled for month blood collection in multivariable analyses to minimise confounding bias. The FFQ did not collect portion sizes, so we were only able to assess frequency of food item consumption, not overall intakes or contributions of country and market foods to overall vitamin D intake. We did not collect information on intake of supplements, which may have acted as an uncontrolled confounder in multivariable analyses. Finally, there is increasing recognition that new vitamin D biomarkers, including metabolites (for example, 3-epi-25(OH) D3, 24R,25(OH)2D3 and vitamin D-binding protein), may be important to assessing vitamin D status and health implications^(53)^. Authors have therefore stressed the need to measure such compounds in health surveys^(54)^. While we measured only total serum vitamin D levels, future research conducted with Inuit communities should also consider measuring other vitamin D biomarkers. Notwithstanding these limitations, our analysis makes important contributions to knowledge on the prevalence and factors associated with vitamin D inadequacy and deficiency among Inuit living in Nunavik.

## Conclusion

Living in northern regions poses challenges to vitamin D homoeostasis. Despite this, just under one-third of Nunavimmiut adults (Inuit living in Nunavik, northern Québec) exhibit vitamin D insufficiency or deficiency, which is slightly lower than the Canadian average and substantially lower than other Inuit populations across Inuit Nunangat assessed in 2007–2009. In this cross-sectional study of 1,155 Nunavimmiut, serum 25(OH)D concentrations increased with older age, higher education, lower latitudes, practicing traditional harvesting activities and country food consumption, particularly wild fish species. Meanwhile, risk of vitamin D deficiency (serum 25(OH)D < 50 nmol/l) was higher among individuals living in a more northerly communities, those who did not report participating in traditional and harvesting activities and those consuming lesser amounts of country foods. Frequency of wild fish consumption was the only country food that was strongly associated with vitamin D status, underscoring its nutritional importance across Nunavik. It is important that national, regional and local policies and programs are in place to secure harvest, sharing and consumption of nutritious and culturally important country foods like Arctic char and other wild fish species, particularly considering ongoing climate change in the Arctic which impacts the availability, access and quality of fish as food.

## Supporting information

Little et al. supplementary material Little et al. supplementary material

## Supplementary material

For supplementary material accompanying this paper visit https://doi.org/10.1017/S1368980024000491
