# Supplementary material for: Wild fish consumption and latitude as drivers of vitamin D status among Inuit living in Nunavik, northern Québec
Source: Public Health Nutr. 2024 Feb 22;27(1):e81. doi: 10.1017/S1368980024000491 (PMC10966833; doi:10.1017/S1368980024000491)
Supplement: Little et al. supplementary material [file S1368980024000491sup001.docx]

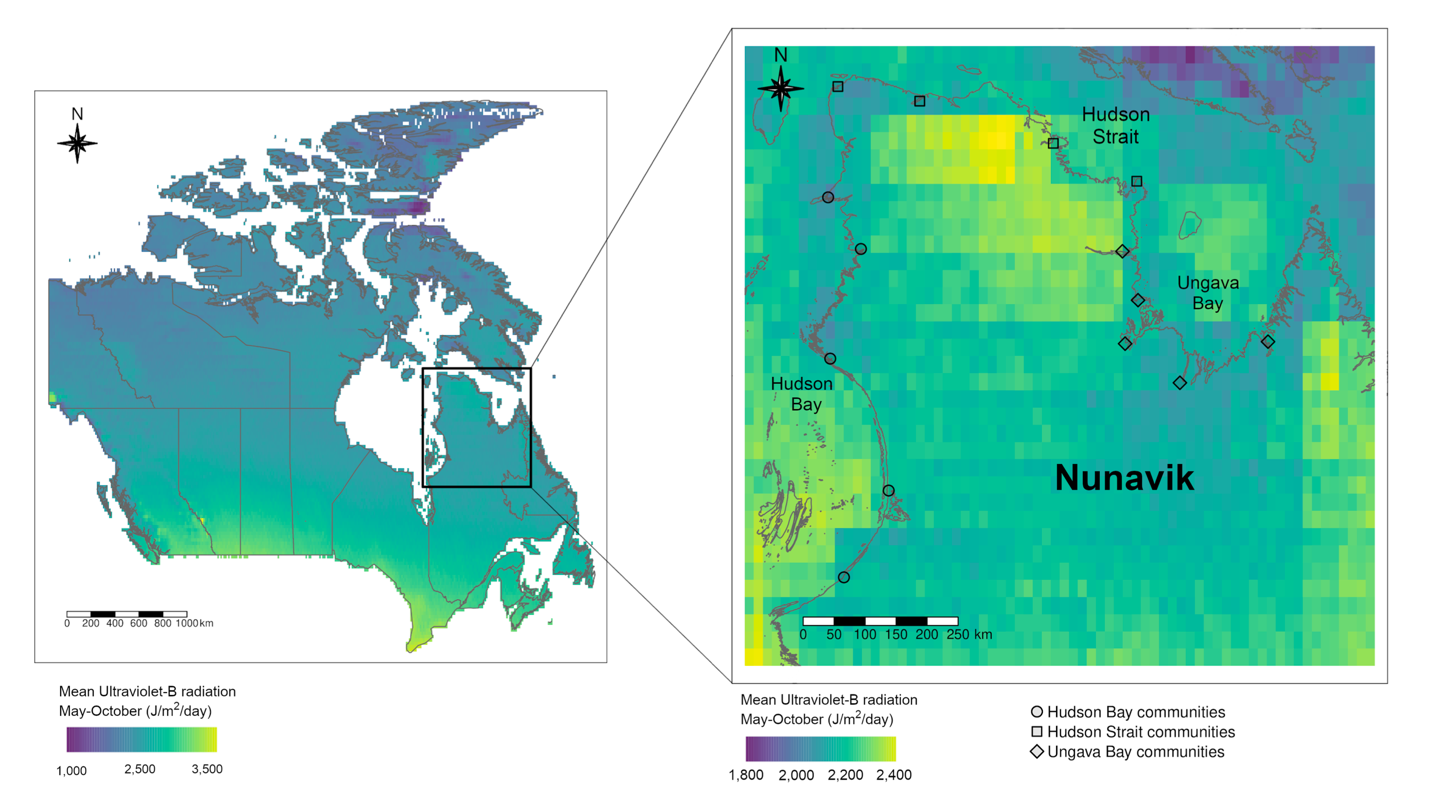


**Supplementary Figure 1**: Mean Ultraviolet-B radiation (J/m^2^/day) for the May - October period for Canada (left) and Nunavik (right). Inferred UV-B values extracted from the glUV dataset (15 arc-minute resolution) from: Beckmann, M., Václavík, T., Manceur, A. M., Šprtová, L., von Wehrden, H., Welk, E., & Cord, A. F. (2014). gl UV: a global UV‐B radiation data set for macroecological studies. *Methods in Ecology and Evolution*, *5*(4), 372-383.
